# Migrant and Refugee Patient Perspectives on Travel and Tuberculosis along the Thailand-Myanmar Border: A Qualitative Study

**DOI:** 10.1371/journal.pone.0160222

**Published:** 2016-08-10

**Authors:** Naomi Tschirhart, Tabitha Sein, Francois Nosten, Angel M. Foster

**Affiliations:** 1 Faculty of Health Sciences, University of Ottawa, Ottawa, Ontario, Canada; 2 Shoklo Malaria Research Unit, Mahidol-Oxford Tropical Medicine Research Unit, Faculty of Tropical Medicine, Mahidol University, Bangkok, Thailand; 3 Centre for Tropical Medicine and Global Health, Nuffield Department of Clinical Medicine, University of Oxford, Oxford, United Kingdom; Agency for Science, Technology and Research - Singapore Immunology Network, SINGAPORE

## Abstract

**Background:**

The Thailand-Myanmar border separates two very different health systems. The healthcare system in eastern Myanmar remains underdeveloped as a result of decades of instability. Comparatively, Tak province, Thailand has more healthcare resources. In this Thai border province government hospitals and non-governmental organizations provide tuberculosis (TB) treatment to migrants and refugees.

**Objectives:**

Our overall study aimed to explore accessibility of TB treatment, TB surveillance and health system responsiveness specific to migrant and refugee populations in Tak province. In this paper, we focus on the perspectives of migrant and refugee TB patients with respect to travel and treatment in Tak province.

**Methods:**

In 2014 we conducted focus group discussions with 61 TB, Tuberculosis and Human Immunodeficiency Virus co-infection, and multidrug-resistant TB patients in Tak province. We analyzed the data for content and themes and documented individual travel trajectories.

**Results and Discussion:**

Migrants are travelling with active TB within the country and between Thailand and Myanmar. Migrants primarily travelled to obtain treatment but two participants reported travelling home to seek family care in Myanmar before returning to Thailand for treatment. Travel, while expensive and arduous, is an adaptive strategy that migrants use to access healthcare.

**Conclusions:**

Migrant’s need for travel points to larger difficulties associated with healthcare access in the border region. Long distance travel with an infectious disease can be seen as an indicator that local healthcare is not available or affordable. These findings suggest that public health officials from both sides of the border should discuss the factors that contribute to travel with active TB and explore potential solutions to mitigate disease transmission in migrant populations.

## Introduction

Tak province is situated in northwestern Thailand and borders Kayin state, Myanmar. The Thailand-Myanmar border separates two very different health systems. The healthcare system in Kayin state and other regions of eastern Myanmar remains underdeveloped as a result of decades of instability and lack of investment in public health infrastructure. In eastern Myanmar healthcare is primarily provided by community-based health organizations and for many people in this region government facilities are unavailable or inaccessible [1]. Comparatively, Tak province has more healthcare resources including a greater number of clinics, hospitals, and health professionals. Tak province can be described as a middle resource setting as compared to a high resource setting like Bangkok that has more specialist hospitals. In Tak province there are five district government hospitals as well as three organizations, Première Urgence—Aide Médicale Internationale (PU-AMI), the Shoklo Malaria Research Unit (SMRU), and the International Organization for Migration (IOM), that provide TB treatment to migrants and refugees. The Thailand-Myanmar border in Tak province is known for its fluidity. The border in Tak province is 500 kilometers in length and the two countries are separated by the Moei River. Migrants can easily swim or take a boat to cross the river. It is not unusual for individuals living in Myanmar to cross over the Thai border to receive treatment at one of the clinics that is dedicated to providing health services for migrants. For this project migrant is defined as an individual who has resided in a foreign country for more than one month or who has crossed a national border to access essential services, irrespective of the causes, voluntary or involuntary and the means, regular or irregular used to migrate. Individuals who have received refugee status are not considered migrants. This description is adapted and modified from a definition used by the International Organization for Migration.

Myanmar and Thailand are both countries with a high burden of people living with tuberculosis (TB), however the prevalence is significantly higher in Myanmar (473/100,000) than in Thailand (149/100,000) [2]. Myanmar is also more heavily burdened with multidrug-resistant tuberculosis (MDR-TB) and it is estimated that 5% of new TB cases and 27% of retreatment cases are multi-drug resistant [2]. Comparatively, in Thailand it is estimated that 2% of new and 19% of retreatment cases are MDR-TB [2]. Tuberculosis has been identified as an important public health issue in Tak province by the Tak provincial public health office [3]. A review of provincial surveillance data for 2006–2011 showed that the majority of TB patients were in Mae Sot district most of whom where non-Thais [3]. Mae Sot is largest urban center along the Thailand-Myanmar border and has been rapidly changing from a small border town to a large special economic zone. A 2007 pilot study also found that the burden of TB in Tak province is higher among migrant populations as 65% of TB patients were non-Thais [4].

In Tak province treatment for TB is provided by both government hospitals and non-governmental organizations (NGOs). Migrants can register and pay into Thailand’s Compulsory Migrant Health Insurance scheme which is a form of insurance providing coverage for healthcare at Thai government hospitals inclusive of TB treatment. Non-Thais without insurance can pay out of pocket for services at the government hospitals but most low waged migrants cannot afford the treatment fees. TB treatment at the Mae Sot Hospital is non-residential and at the time of our study World Vision Thailand was providing Directly Observed Treatment (DOT) to migrants living in urban Mae Sot, Thailand. DOT was not provided to migrant TB patients who were living in Myanmar and crossing the border to get treatment at the Thai government hospital. In Tak province PU-AMI and SMRU have set up residential TB treatment programs specifically for migrants and refugees who are not eligible to receive low cost treatment from Thai government hospitals. PU-AMI provides TB and Tuberculosis and Human Immunodeficiency Virus co-infection (TB/HIV) treatment in the refugee camps. SMRU treats migrants and refugees with TB, TB/HIV and is the only NGO in Tak province that provides MDR-TB treatment to this population. Both PU-AMI and SMRU have residential TB villages where patients can stay during treatment and receive daily DOT. The TB villages are overseen by TB doctors and are staffed by medics who are migrants and refugees from Myanmar. IOM gives TB treatment specifically to refugees who are relocating to a third country. There are linkages between the treatment providers and in 2012 SMRU, PU-AMI, IOM and the Tak provincial public health office, created the Tuberculosis Tak Border Initiative (TTBI) as a mechanism to collaboratively address the TB burden in Tak province [5]. The five district hospitals are TTBI implementing partners and received funding to provide TB treatment for migrants through a grant from the United Kingdom’s Department for International Development and the European Union. World Vision Thailand does not provide clinical treatment for TB but plays an important role by helping to refer symptomatic patients and providing supportive care and DOT for migrant patients who were treated at the Thai government hospital.

## Methods

Our interdisciplinary team conducted substantive data collection in the summer and fall of 2014 with an additional data collection period in December 2015. In brief our overall study included key informant interviews with treatment providers and public health officials (n = 13), 15 focus group discussions (FGD) with TB patients (n = 61) and non-patients (n = 31), and a survey of community health volunteers (n = 101). We designed this multi-method research project to explore access to TB treatment among refugee and migrant populations in Tak province. Our primary research objective for the overarching research project was to identify how migrants and refugees access TB, TB/HIV and MDR-TB treatment in Tak province. During the initial analysis we found that migrants are travelling long distances within Thailand and across the border to and from Myanmar with active TB. In this paper we focus on the perspectives and experiences of migrant and refugee patients with respect to travel and treatment.

### Data collection: focus group discussions

We held FGD with migrants and refugees who were living or seeking healthcare in Tak province. To create a safe space for discussion we attempted to create homogenous focus groups with participants disaggregated by health status, gender, and migrant or refugee identification [6]. We prioritized holding different FGD based on health status as we anticipated participants would have specific challenges related to differential availability of TB, TB/HIV and MDR-TB care in Tak province. We conducted eleven FGDs for TB, MDR-TB, and TB/HIV patients and four FGD with non-patients. As treatment providers often provided care to both migrant and refugees, some FGD had both refugees and migrants. We do not anticipate that this affects the study as participants gave their personal histories and we used this information to differentiate between refugees and migrants. In total, sixty-one patients with TB, TB/HIV or MDR-TB participated in the FGDs. Our research participants had diverse backgrounds including migrant workers who were living in Thailand, migrants who had travelled from Myanmar for treatment, and residents living in a camp along the border. We present participant information in Table 1. While our non-patient migrant FGD participants indicated that they had challenges to access healthcare generally, we have not included their data in this particular article as they did not travel with TB. These migrants who were living in Mae Sot, Thailand indicated that they had challenges accessing healthcare related to their legal status, however they had less difficulty than migrants who had travelled from Myanmar or other provinces as free healthcare is readily available for migrants in Mae Sot at the Mae Tao Clinic.

**Table 1:** Composition of TB Patient Focus Groups.

FGD	Location	Type	Number of participants	Description
1	Mae La TB village	Men with TB	6	Refugees and Migrants
2	Mae La TB village	A man and woman with active TB	2	Refugees
3	Mae La TB village	Women with TB	5	Refugees and migrants
4	SMRU TB village	Women with TB	5	Migrants
5	SMRU TB village	Men with TB	7	Migrants
6	SMRU TB village	Women with TB/HIV	7	Migrants
7	SMRU TB village	Men with TB/HIV	8	Migrants
8	SMRU TB village	Women with MDRTB	6	Refugees and migrants
9	SMRU TB village	Men with MDRTB	7	Refugee and migrants
10	Mae Sot Hospital	Women with TB	3	Migrants
11	Mae Sot Hospital	Women and Men with TB	5	Migrants

We recruited patients from a TB treatment facility run by SMRU, a TB clinic at the Mae Sot Hospital, and through a refugee camp healthcare centre that is operated by Première Urgence-Aide Médicale Internationale. Clinicians and clinic staff informed eligible individuals of the study, explained that participation is voluntary and clarified that the decision to participate would not affect their right to receive medical care. Prospective participants who indicated that they were interested were informed of the location and time of the FGD. As this is a highly mobile population residing in a large geographic area spanning two countries, we know from previous experience that it is beneficial to have the FGD shortly after the recruitment period. In the case of this research project, we conducted the focus groups within one to two days of recruitment.

We held the FGDs at the Mae Sot Hospital, the Mae La refugee camp TB village, the SMRU TB village, and at two community health posts in Mae Sot. Each place where we held a discussion was quiet, secure and separate from where clinical and counseling services were provided. Participants received 150 baht (approximately USD 4) as a reimbursement for travel expenses as well as food to take home. All participants consented for the FGDs to be audio recorded. After obtaining consent, NT conducted the focus groups with the assistance of a Burmese-speaking interpreter and TS, a co-facilitator and Karen-speaking interpreter. Focus group discussions lasted approximately 45 minutes and explored participants’ pathway to treatment as well as the resources that enabled them to continue treatment.

### Data analysis

TS transcribed the Karen and Burmese audio recordings into English. We then analyzed the transcripts and coded the data in Nvivo using *a priori* (predetermined) and emergent codes. NT then conducted second and third level analysis to categorize content and identify themes. During the analysis we identified travel with TB as an emergent theme, which we explore in detail in this paper. With the aim of further investigating travel, NT went back to the raw data and documented the individual journeys by identifying the geographical locations that participants described visiting during their quest for TB treatment. Where trajectory information was unavailable we still included any travel related contributions in the thematic analysis. We also used Google My Maps to calculate approximate distances that the patients travelled to gain access to treatment and to map travel trajectories. We returned to Mae Sot in Tak province in June 2014 to present our preliminary findings to stakeholders many of whom participated in the research as key informants. These presentations and the subsequent discussions served as a member checking exercise that allowed us to enhance the quality and “trustworthiness” of our findings [7]. In this paper we have organized the results by theme.

### Ethics

We received research approval for this study from the Health Sciences and Sciences Research Ethics Board at the University of Ottawa (File #H02-14-08), the Oxford Tropical Research Ethics Committee at the University of Oxford (538–14) and the Tak Provincial Public Health Office (TK 1/2557). To protect the identity of the participants we have masked all personally identifying data and have used pseudonyms. Pseudonyms are commonly used in qualitative research to mask the identity of the participant.

## Results

We collected partial or full travel trajectory information from forty-two migrant patients. Some of the migrant patients were living in Tak province, while others resided in Myanmar or Bangkok and had travelled to get treatment. Trajectory information was missing for eleven migrant patients and we did not collect it from eight refugees. Comparative to the migrant population in this study, refugees were less mobile and were living inside a gated refugee camp. As TB treatment is available inside the refugee camp where we conducted the FGDs, most refugee patients did not need to travel outside of the camp boundaries to get treatment. For refugee patients with MDR-TB, the health care provider PU-AMI organized free transportation outside the camp to the SMRU clinic. Refugee participants did not embark on independent travel with TB and reported limited challenges accessing treatment. In the results section we explore the experiences of migrant patients.

### Migrants are travelling with active TB

During several FGDs participants from Myanmar described travelling with symptoms of active tuberculosis. In some cases individuals knew or suspected that they had TB based on a past clinical TB diagnosis. Of the forty-two patients that travelled it is likely that most were infectious as all of them had TB that was either untreated or not responding to treatment. The exception is one patient who had bone TB which is not infectious. Our FGD participants shared their own stories of travel. Their journeys began in locations with varying levels of healthcare resources and ended with TB treatment in Tak province. Some travelled from higher healthcare resource settings like Bangkok and others came from villages in rural Myanmar with low healthcare resources. Migrants’ travel to TB providers in Tak province originated within the province (n = 15), from Myanmar (n = 21), and from Bangkok (n = 2). In addition four patients crossed the Thailand-Myanmar border twice by travelling from Bangkok, Thailand to Myanmar and then back to Tak province. FGD participants used their social networks to locate and gain access to TB treatment. Patients and their family members asked neighbors, friends and healthcare workers where they could get treatment. In one case a woman in Thaton, Myanmar met two monks who were travelling to Thailand to get healthcare and asked permission to follow them. Several participants came to stay with their family member in the refugee camp in order to access health services.

### Migrants primarily travelled to access TB treatment

I am from Rangoon [Yangon], I came here just to get treatment. I arrived in Thailand over a year ago and spent 2 months in Mae La [Refugee] Camp. I have been taking MDR-TB medication for 13 months now. I received TB treatment at Rangoon for only one month and I was not feeling better. They asked me to stay at the hospital and tested my sputum for bacteria so many times. Then they found out that I had MDR-TB. The medication for MDR-TB was very limited so I had to wait for the new medication to come. Myat Noe, female migrant MDR-TB patient

Most FGD participants travelled exclusively to get treatment. Participants came from Myawaddy and Koko which are directly across the border from Tak province as well as further inside Myanmar including Yangon (440 km) and Mawlamyine (180 km) specifically to seek TB care. Participants who were living in Thailand, both Bangkok (500km) and Tak province, also travelled primarily to get TB treatment for themselves. The two exceptions were patients who initially arrived at a TB clinic in Tak province in the role of a support person accompanying a TB patient. In this case the individuals undertook travel to get treatment for their loved one not realizing that they also had TB.

Participants described travel as an expensive and arduous and explained that they needed to travel because they could not appropriate and effective care locally. Treatment failure, treatment unavailability and treatment unaffordability influenced participants’ decisions to travel. Referrals from individuals who had heard of free health services available in Tak province also factored into the decision to travel.

FGD participants who had received unsuccessful TB treatment explained that they were not getting better and subsequently decided to travel to seek care. Reports of previous treatment failure were more common among patients with MDR-TB than drug susceptible TB or TB/HIV. Some of the MDR-TB patients described knowing that they had drug resistant TB while others only knew that they were unwell and that previous treatment didn’t work.

**Fig 1:**
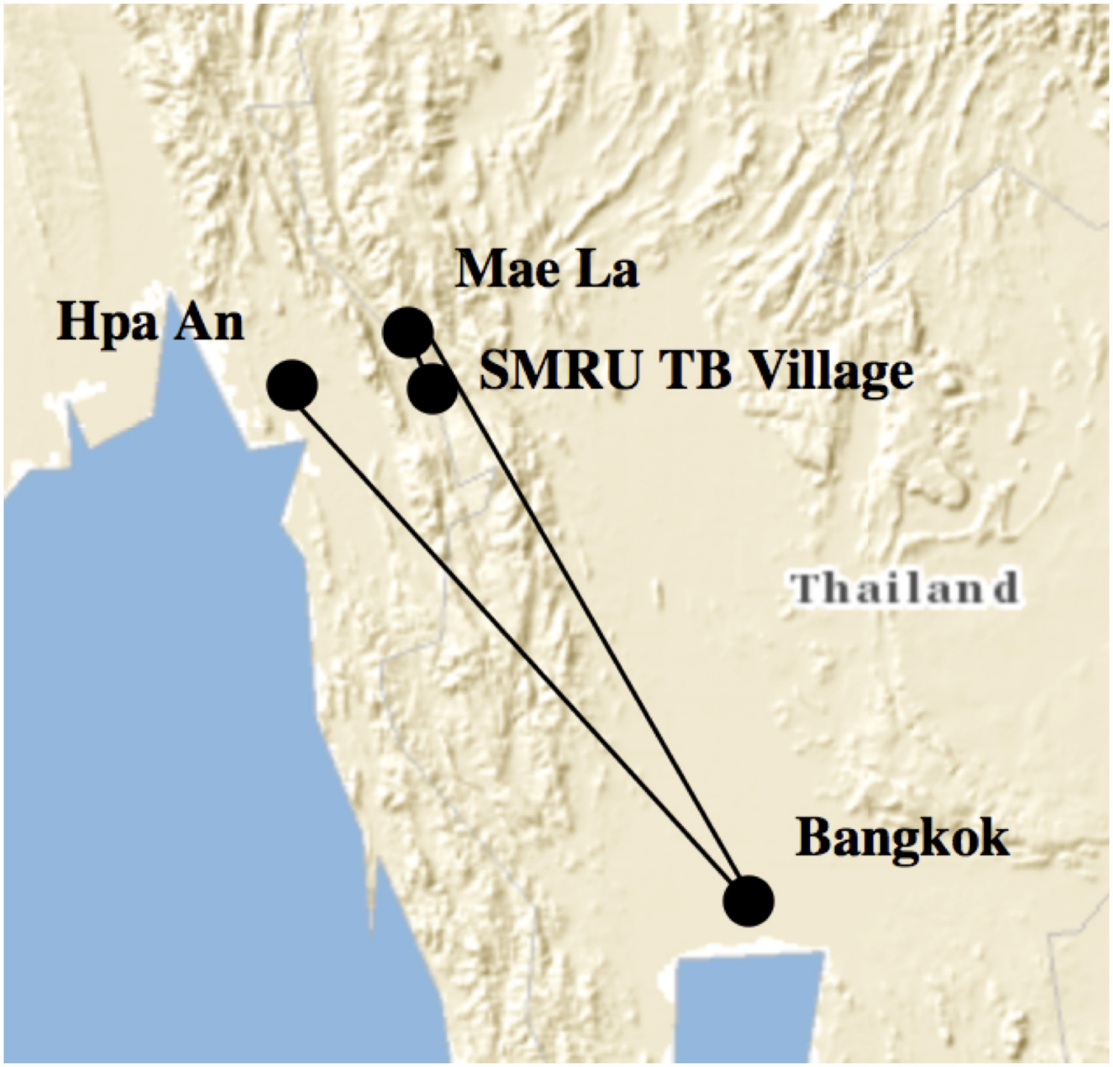
MDR-TB patient’s travel trajectory from Hpa An, Myanmar to seek TB treatment in Tak province. Lines indicate travel pattern. Image similar but not identical to original created using the USGS National Map Viewer and therefore used for illustrative purposes only.

Here we explore the trajectory of one MDR-TB patient. Fig 1 traces the 1214 kilometer travel trajectory of Saw Paul, a Karen man from the original location where he sought treatment to the SMRU TB in Tak province village where he received treatment for MDR-TB approximately two years later. In 2011 he travelled from Kaw Ler village to Hpa-An which is a mid-sized town in south-eastern Myanmar to receive his first seven month treatment. Unfortunately it was not effective and he got a second seven month course of medication in Hpa-An. After two rounds of unsuccessful treatment he went with his daughter to Bangkok (654 km) and purchased TB medication from a pharmacy and treated himself for nine months. Saw Paul still didn’t feel better so he travelled 560km north to the Mae La refugee camp where he received treatment before being referred to the nearby SMRU TB village (61 km) for MDR-TB treatment.

Where treatment was available, some participants who were living in Myanmar and Bangkok when they fell ill explained that they could not afford to seek treatment locally due to high treatment costs. A second reason that participants travelled to get treatment is that the treatment that was available closest to them was unaffordable. Patients who were living inside Myanmar and those who were working in Bangkok when they fell ill explained that they could not afford to seek treatment locally due to high treatment costs. Myanmar has a national health care system where costs are shared between the patient and the government. In Bangkok, migrants who have not registered and paid to join the government health insurance scheme must pay out of pocket for health services.

In searching for a place to get treatment patients relied on advice from friends and family members. Mae Tao Clinic (MTC), a well known clinic in Tak province that provides health services to migrants, was identified as a preferred treatment centre. MTC tests symptomatic patients for TB and refers clients to another provider in Tak province for TB treatment. While most FGD participants initiated their own travel plans after consulting with friends and family, three patients were referred from outside Tak province. A physician in Bangkok told one patient to travel to MTC for treatment, a second patient was referred to Mae Sot hospital from Myawaddy Hospital for bone TB treatment due to insufficient medication and medical equipment and a third patient with MDR-TB was referred from an IOM clinic in Mawlamyine, Mon State, Myanmar.

#### The need for family support can motivate travel with TB

While most of the FGD participants travelled with TB exclusively to seek treatment, four patients took a circuitous route which involved travel home to Myanmar from Thailand before returning to Thailand for treatment. Two of the participants described why they took this route. Both women were working in Bangkok when they fell ill with TB and subsequently decided to go home to Myanmar so their families could take care of them. One woman didn’t have the legal documentation required to obtain low cost healthcare at Thai government hospitals and the second had the required insurance but didn’t want to burden her husband with the responsibility to take care of her during a second course of TB treatment. Both women described that when they returned to Myanmar they found that treatment was unavailable close to their family home and that travel and treatment costs were unaffordable. They subsequently decided to return to Tak province for TB treatment.

### Travel for migrants is arduous

Of course I have no money and so I walk to die even though I have no energy. Aung, male TB patient.

FGD research participants described arriving at the clinic by different means of transportation. Some walked but most took shared transportation such as cars or buses. Patients who were referred from clinics within Tak province received free transportation from the health service provider. Travel can be expensive and travel costs were identified as a barrier to accessing treatment. Some participants borrowed money to be able to make the trip. For migrants without proper documentation travel can be logistically challenging and significantly more expensive. Undocumented migrants described paying higher rates to take routes to avoid the police. Many participants expressed fear of being stopped by the police and being asked to pay a lump sum or face deportation. In selecting where to get treatment migrants described making comparative calculations of the total costs inclusive of treatment and transportation fees. Eka a migrant woman from nearby Myawaddy which is directly across the border articulated the comparative financial implications, “we have to pay for the transportation fees only, not the treatment fees so we choose to come here. In Burma we have to pay both”. For patients like Eka in Myawaddy it is closer to travel to Mae Sot in Tak province Thailand (less than 10km) than further inland to Mawlamyine (172km).

## Discussion

Cross border travel for the purpose of seeking healthcare along the Thailand-Myanmar border has been well documented in the literature [4]. However less is known about the rationale for travel from the patient perspective. Our study contributes new knowledge to this area. We found that migrants are travelling long distances with symptoms of active TB to seek medical treatment or to return home to receive care from their family while they are ill.

### Travel is an adaptive strategy used to access TB treatment

Migrants described travel as expensive and arduous. Yet despite the associated difficulties including police bribes and the risk of deportation, travel was often deemed necessary. Our findings suggest that the need to travel emerges from contextual factors that shape the environment where migrants live and work. Specifically along the Thailand-Myanmar border the decision to travel is influenced by synergistic interactions between different social determinants of health including migration, the healthcare system and material circumstances. Our research supports the notion that migration itself is a social determinant of health given the social inequalities migrants face and the health impacts of the migration progress [8]. We found that migrants’ legal status and personal finances influenced where they are eligible to receive healthcare. For example several patients travelled to the capital of Thailand, which has extensive health care services, then to Tak province as they were not eligible to receive free or low cost care in the capital Bangkok because they were not registered in the Thai government’s migrant health insurance scheme. Unaffordability of health services in Myanmar also contributed to patient’s decisions to travel to Tak province where they could receive free or low cost treatment. Our results suggest that availability of care is an important consideration. Where care is not available locally travel becomes necessary. This theme was especially prominent for MDR-TB patients who often travelled to several locations to seek treatment before coming to Tak province. We expect that the continual search and the associated extensive travel were financially catastrophic for patients. Daily waged migrants who participated in this research described travel costs that often exceeded their daily wage even for those that were travelling shorter distances from within Tak province. From a population health perspective, travel can be seen as an adaptive strategy for migrants who are seeking to gain access to TB treatment. We observe that migrants’ decision to travel is linked to the high individual and familial costs associated with TB in their specific context. For example, migrants living in locations where free TB treatment was not available indicated concerns about treatment costs in addition to lost wages. Migrants described weighing the decision to travel and comparing it against their other options. In using their social networks to find out where they can get treatment they demonstrate resiliency and resourcefulness. Finally the existence of TB treatment provided by NGOs in Tak province allows migrants the possibility to access free treatment through travel. Our research supports the notion that migrants will continue to come to access healthcare in Tak province until healthcare accessibility improves in Myanmar [4].

### Going home with TB

In conducting this research we did not expect to find four cases of migrants who travelled with TB from Bangkok, Thailand to Myanmar. This finding is particularly surprising in the case of one woman who had health insurance and could access healthcare in Thailand’s capital city for a nominal fee but chose to return to Myanmar so that her family could take care of her during her illness. Given that only two participants indicated that they were travelling home specifically to be with their family, these cases serve as an anomaly, albeit an important one. Our limited data suggests that social support and care is important during treatment and compliment the findings of a study from India that documented migrants moving back to their home village following TB illness [9]. Further research may be necessary to explore TB treatment, social support and decisions to return home among patients from Myanmar. Additional insight into the decision to travel while ill from a high healthcare resource setting to a low resource setting may be especially valuable. We anticipate that these decisions are likely multi-dimensional and may take into account social support as well as treatment and living costs.

### Public health implications

Patients who participated in our project did not articulate concerns about TB transmission during travel, however travel with active tuberculosis on public transport has broader health implications. In our study we identified forty-one patients who travelled with TB that was untreated or not responding to treatment. People with active TB who are travelling short or long distances on shared public transportation can potentially expose other passengers to TB bacteria. People travelling across borders with MDR-TB is even more concerning as MDR-TB is more difficult and expensive to treat than drug susceptible TB. Additionally, inadequately treated MDR-TB in individuals with HIV has been shown to be highly infectious [10]. In this study migrants described travelling by shared car, bus and boat to reach health services in Tak province. Given the small number of studies conducted of TB transmission on public transport, it is difficult to quantify the risk and to define the public health implications [11]. However, other work suggests transmission is possible and in a study from Spain a single six hour bus ride with one TB case led to a transmission rate of 21/53 (39.6%) and five contacts subsequently developed active disease [11]. Crowding, duration and cumulative exposure increase the risk of TB transmission [11].

In high TB burden contexts like Thailand and Myanmar it is possible that an individual patient was exposed to TB bacteria multiple times prior to the disease. Multiple exposures, a long incubation period and the challenge of tracing cases across national borders make it is difficult to attribute TB disease to one specific ride on public transport. Our data suggests migrants with TB may undertake multiple trips in order to get effective treatment. Migrants experienced delays in accessing treatment due to availability and affordability of care. Delayed TB diagnosis is common among migrants [12]. Treatment delays extend the length of time the individual is infectious and contribute to transmission [13]. Delayed treatment further perpetuates the cycle of TB infection and disease among the migrant community. Lack of accessible and appropriate TB care may also contribute to patient’s decisions to self manage their own TB regimens. While only one participant in our study purchased their TB medication from a pharmacy, self-treatment is an urgent threat for drug resistance and is an important public health concern in this region.

### Limitations

One of the limitations that we identified is that this research project only collected information from individuals who successfully accessed treatment. We know much less about individuals with TB who have not been able to get treatment. An additional limitation is the temporal nature of the study. Our team conducted the FGDs in September and October 2014. Access to healthcare for migrants continues to evolve as policies change. As such our results should be interpreted with reference to the specific temporal period. While we conducted our recruitment with the assistance of clinicians and clinic staff at TB facilities, participants were informed that their participation was voluntary and we do not anticipate that this has any implications for our findings. The results from this qualitative research project are not generalizable to all migrants and refugees on the Thailand-Myanmar border. However, we hope our findings on rationale for travel may probe additional research along the border and in other regions.

## Conclusions

For migrants, traveling long distances is costly and logistically challenging. In choosing to travel individuals have often exhausted other options for getting medical treatment and supportive care. Migrants explained that they travelled with active TB either to get treatment or to return home to their family. Travelling with active TB on public transport has an inherent risk for TB transmission, however we identified travel as an adaptive strategy that migrants use to gain access to healthcare. Our finding that cost and unavailability of TB and MDR-TB treatment contribute to mobility across provincial and national boundaries has important implications for public health officials in the region. The results point to larger difficulties associated with healthcare access. Long distance travel with an infectious disease can be seen as an indicator that local healthcare is not available or affordable. Public health officials from both sides of the border could discuss the factors that contribute to travel with active TB and explore potential solutions to mitigate disease transmission in migrant populations.

## Abbreviations

DOT: Directly observed treatment
FGD: Focus group discussion
IOM: International Organization for Migration
NGO: Non-governmental organization
SMRU: Shoklo Malaria Research Unit
TB: Tuberculosis
MDR-TB: Multidrug-resistant tuberculosis
PU-AMI: Première Urgence—Aide Médicale Internationale

## References

[1] Health Information Systems Working Group. The long road to recovery: ethnic and community-based health organizations leading the way to better health in eastern Burma. [Internet]. (n.p.): Health Information Systems Working Group;. 2015 [cited 2015 Jul 27]. Available: http://cpintl.org/sites/default/files/bookPdf/The%20Long%20Road%20to%20Recovery%202015_Eng%20%281%29.pdf.

[2] World Health Organization. Annex 2 country profiles. In: Global Tuberculosis Report 2014. Geneva: World Health Organization; 2014.

[3] Iemrod K, Kavinum S. Strategies for development of the tuberculosis control program in Tak province. The Public Health Journal of Burapha University. 2015;10:2–14. Thai.

[4] Hemhongsa P, Tasaneeyapan T, Swaddiwudhipong W, Danyuttapolchai J, Pisuttakoon K, Rienthong S, et al TB, HIV-associated TB and multidrug-resistant TB on Thailand’s border with Myanmar, 2006–2007. Trop Med Int Health. 2008; 13(10):1288–96. 10.1111/j.1365-3156.2008.02139.x 18721186

[5] Shoklo Malaria Research Unit. TB Field [Internet]. Thailand: Shoklo Malaria Research Unit;. c2014—[cited 2015 Sep 30]. Available: http://www.shoklo-unit.com/index.php/smru/tb.

[6] Kamberelis G, Dimitraidis G. Focus groups: strategic articulations of pedagogy, politics, and inquiry In: Denzin NK, Lincoln YS, editors. The Sage handbook of qualitative research. Thousand Oakes: Sage; 2005 pp.887–907.

[7] Guba EG, Lincoln YS. Competing paradigms in qualitative research In: Denzin NK, Lincoln YS, editors. Handbook of qualitative research. Thousand Oaks: Sage; 1994 pp 105–117.

[8] Davies A, Basten A, Frattini C. Migration: a social determinant of migrant’s health. Eurohealth. 2006;16(1):10–12.

[9] Jayachandran V. A case study on tuberculosis treatment defaulters in Delhi: weak health links of the community with the public sector, unsupported migrants and some misconceptions. Ann Trop Med Public Health. 2014;7(2):124–129.

[10] Escombe AR, Moore DAJ, Gilman RH, Pan W, Navincopa M, Ticona E, et al The infectiousness of Tuberculosis patients coinfected with HIV. PLoS Med. 2008; 5(9):e188 10.1371/journal.pmed.0050188 18798687 PMC2535657

[11] Mohr O, Askar M, Schink S, Eckmanns T, Krause G, Poggensee G. Evidence for airborne infectious disease transmission in public ground transport—a literature review. Euro Surveill. 2012;17(35):1–11. 22958608

[12] Abarca Tomás B, Pell C, Bueno Cavanillas A, Guillén Solvas J, Pool R, Roura M.Tuberculosis in migrant populations. A systematic review of the qualitative literature. PLoS ONE. 2013; 10.1371/journal.pone.0082440 PMC3857814 24349284

[13] Narasimhan P, Wood J, MacIntyre CR, Mathai D. Risk factors for tuberculosis. Pulmonary Medicine. 2013; 10.1155/2013/828939 PMC3583136 23476764

